# The Relationship between Sarcopenia and Injury Events: A Systematic Review and Meta-Analysis of 98,754 Older Adults

**DOI:** 10.3390/jcm11216474

**Published:** 2022-10-31

**Authors:** Yu-Chen Su, Shu-Fang Chang, Hsiao-Chi Tsai

**Affiliations:** 1 Department of Nursing, College of Nursing, National Taipei University of Nursing and Health Sciences, 365 Ming Te Road, Pei-Tou, Taipei 112303, Taiwan; 110089@ctcn.edu.tw; 2 Cardinal Tien Hospital, No.15, Chezi Rd., Xindian Dist., New Taipei City 112303, Taiwan

**Keywords:** meta-analysis, negative health effects, sarcopenia, systematic review

## Abstract

The main purpose of this study was to investigate the relationship between sarcopenia and injury events (falls, fractures, hospitalization, disability, and death). This study systemically searched the literature from Embase, PubMed, MEDLINE, CINAHL, and Cochrane Library and analyzed the collected literature using the random effects model to demonstrate the relationship between sarcopenia and injury events. This study followed the guidelines of the Preferred Reporting Items for Systematic Reviews and Meta-Analyses (PRISMA) and collected a total of 35 prospective studies. The results showed that, when compared to robust individuals, the risk of injury events for older individuals with sarcopenia was significantly higher for fall (HR = 1.93, CI: 1.29–2.87), fractures (HR = 2.25, CI: 1.77–2.86), hospital admissions (HR = 1.52, CI: 1.28–1.80), disability (HR = 2.74, CI: 1.73–4.34), and death (HR = 2.09, CI: 1.71–2.55). In consideration of the negative impact of sarcopenia on the subsequent health of older adults, professional nursing personnel should assess older adults for sarcopenia as early as possible and propose relevant care policies to further reduce negative health impacts.

## 1. Introduction

With the worldwide trend of aging, population aging has begun to attract global attention. The World Health Organization [1] estimated that from 2016 to 2100, the population over the age of 60 globally will rapidly increase from 0.9 billion to 3.2 billion. For the above reasons, increasing attention must be paid to older adults’ care. As age increases, the rate of degeneration becomes faster, and after the age of 70, it decreases by about 15% per decade [2,3]. Due to the gradual decrease in muscle strength and mobility among older adults, the risks of negative outcomes are increased, resulting in the loss of the ability to live independently. According to a past study, half of the older population over the age of 80 experiences inconvenient mobility, disability, and poor quality of life [4,5,6,7].

Sarcopenia is regarded as a sign of functional deterioration in older adults as well as an intermediate stage between life independence and death pre-sarcopenia refers to low muscle mass, sarcopenia refers to low muscle mass in combination with weak muscle strength or poor physical performance, and severe sarcopenia refers to the decline of all three of the above [8]. The WHO indicates that sarcopenia has become an important factor affecting the successful aging of older adults [9]. The study indicated that starting roughly from the age of 30, the muscles of the human body gradually degenerate and decrease at a rate of 3–8% every 10 years. Studies associated with sarcopenia have shown that the prevalence of sarcopenia among older adults in the United States is 9.6% and that of pre-sarcopenia is 47% [10]. The prevalence of sarcopenia among older adults in the U.K. is 14% [11], while that in Europe is 2.6%, and the prevalence of pre-sarcopenia in Europe is 38.8% [12]. Biritwum et al. [13] discovered that the proportion of older adults over the age of 50 in six countries, including China, Ghana, India, Mexico, Russia, and South Africa, accounts for 43% of the global population of older adults. Researchers have even indicated that the risk of death in older adults with sarcopenia is higher than that of those without it [14]. Moreover, it has been estimated that the medical expenses caused by sarcopenia per year in the United States are approximately USD 26.2 billion [15].

Geriatric experts generally define sarcopenia as an increase in vulnerability and a decrease in the ability to maintain dynamic balance [8,16,17,18,19,20]. Scholars have indicated that sarcopenia can easily lead to a decline in overall health and multiple organs in older adults [14,21,22,23,24,25]. However, there are few studies performing a comprehensive investigation on the injury events of sarcopenia on individuals’ overall health. As a result, it is necessary to conduct a systematic literature review and meta-analysis to further investigate the issues mentioned above. Evidence-based study results could help medical and nursing personnel further understand the injury events of sarcopenia on the subsequent health of older adults to reduce the occurrence of injury events induced by sarcopenia.

### Aims

The main purpose of this study was to investigate the relationship between sarcopenia and injury events (falls, fractures, hospitalization, disability, and death).

## 2. Methods

The main purpose of this study was to investigate the relationship between sarcopenia and injury events (falls, fractures, hospitalization, disability, and death). This study systemically searched the literature from Embase, PubMed, MEDLINE, CINAHL, and Cochrane Library. This study followed the guidelines of the Preferred Reporting Items for Systematic Reviews and Meta-Analyses (PRISMA) [26].

### 2.1. Sarcopenia Assessment

The assessment indicators of sarcopenia include the assessment of sarcopenia proposed by the European Working Group on Sarcopenia in Older People (EWGSOP) in 2020 [27], which proposed common guidelines on the clinical definition, diagnostic criteria, international disease classification code and treatment guidance for sarcopenia. According to the definition proposed by EWGSOP, pre-sarcopenia refers to low muscle mass, sarcopenia refers to low muscle mass in combination with weak muscle strength or poor physical performance, and severe sarcopenia refers to the decline of all three of the above. In addition, the Asian Working Group for Sarcopenia (AWGS) also proposed an Asian sarcopenia assessment consensus version. AWGS defined sarcopenia as low muscle mass and low muscle strength accompanied by low physical performance. It also proposed an Asian version of the cut-point indicator [28].

### 2.2. Data Sources and Search Strategy

The researchers conducted a systematic literature search on Embase, PubMed, MEDLINE, CINAHL, and Cochrane Library. The literature search ended in April 2022. The keywords searched included “sarcopenia”, “muscular atrophy”, “fall”, “fracture”, “hospitalization”, “disability”, “mortality”, “older people”, “older adults”, “geriatric”, and “senior”.

### 2.3. Inclusion and Exclusion Criteria

The inclusion criteria of this study were: (1) studies based on a prospective cohort design; (2) older adults research participants over the age of 65; (3) assessment of the differences between sarcopenia and negative health-related events (falls, fractures, hospitalization, disability, and death) in the research samples; (4) a confidence interval (CI) of 95%; and (5) studies published in English with full text. The exclusion criteria were literature review papers, letters to editors, chapters of books, Master’s and PhD theses, and experimental interventional studies.

### 2.4. Data Extraction

The two researchers, respectively, reviewed and extracted the searched data, and then presented the data on the research subjects (including gender), sample size, follow-up time, and assessment tools included in various studies to further analyze the prediction of sarcopenia for the subsequent occurrence of negative health-related events. In case of any inconsistency between the two researchers during data extraction, a third data reviewer was invited to perform the review.

### 2.5. Quality Assessment

We used the Newcastle-Ottawa Scale (NOS) to evaluate the prospective cohort studies for selection, comparability, and assessment of outcome [29], with a maximum score of 9. Scores ≥ 7 demonstrated a low risk of bias, scores of 4–6 indicated a moderate risk of bias, and scores < 4 showed a high risk of bias.

### 2.6. Statistical Analysis

The calculated hazard ratios (HR) or odd ratios (ORs)of the outcomes were extracted from the included studies. We extracted the HRs or ORs if the authors provided several HRs or ORs with different covariates in the article. We pooled the HRs or ORs using a random effects model that allowed the true effect size to vary across individual studies and assumed that the true underlying effect followed a normal distribution. The heterogeneity of the effect sizes (HRs or ORs) across individual studies was assessed using the *I*² statistics. Data analyses were performed using Comprehensive Meta-Analysis 3 (BioStat Solutions, Inc., Englewood, NJ, USA).

### 2.7. IRB Approval Number

Not applicable. This is a study of systematic review and meta-analysis. Human subject review or compliance (e.g., IRB protocol number) in the manuscript document is not applicable.

## 3. Results

### 3.1. Study Sample

**Figure 1:**
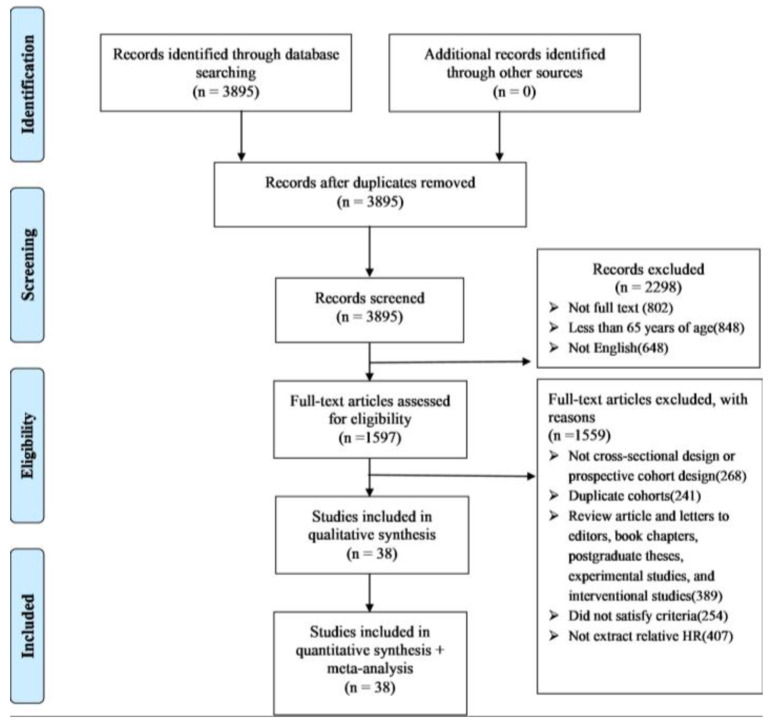
Research flowchart.

Figure 1 depicts the details of the literature review. Among the initial studies identified, we excluded any study that lacked full text, was not in English, and duplicate cohorts and review articles, or that did not satisfy the inclusion criteria. After excluding these studies, we included 38 prospective cohort studies after agreement by the two reviewers. Table 1 summarises the characteristics of these studies for meta-analysis.

### 3.2. Quality Assessment

The studies were scored by NOS, and all of them indicated a low risk of bias; the minimum score was eight, the maximum score was nine, and the average score was 8.8 (Table 2).

### 3.3. Association between Sarcopenia and Injury Events

**Figure 2:**
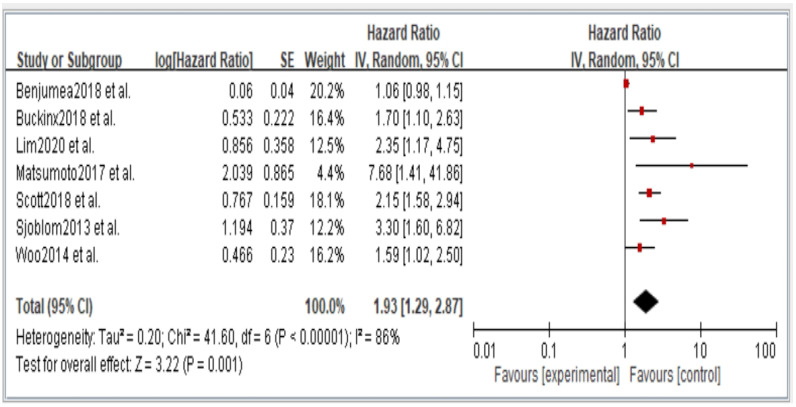
Summary estimates for the sarcopenia status compared to fall outcome.

**Figure 3:**
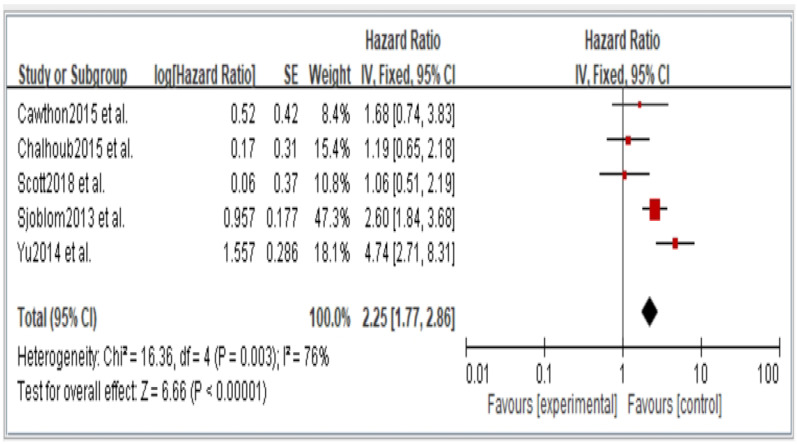
Summary estimates for the sarcopenia status compared to fracture outcome.

**Figure 4:**
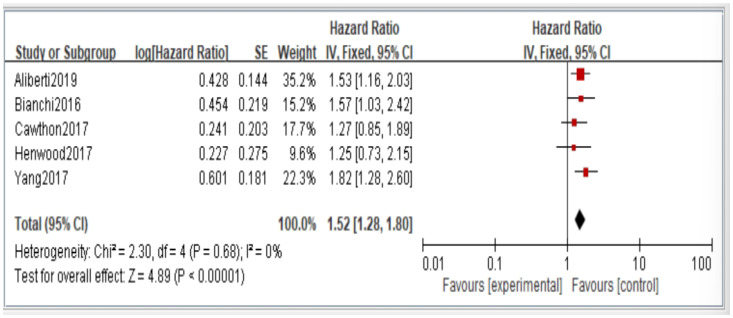
Summary estimates for the sarcopenia status compared to hospitalization outcome.

**Figure 5:**
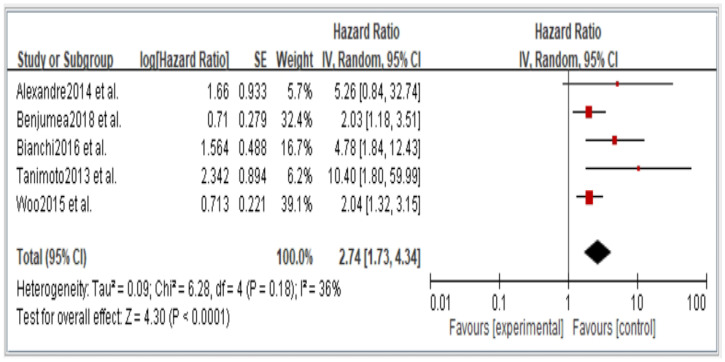
Summary estimates for the sarcopenia status compared to disability outcome.

**Figure 6:**
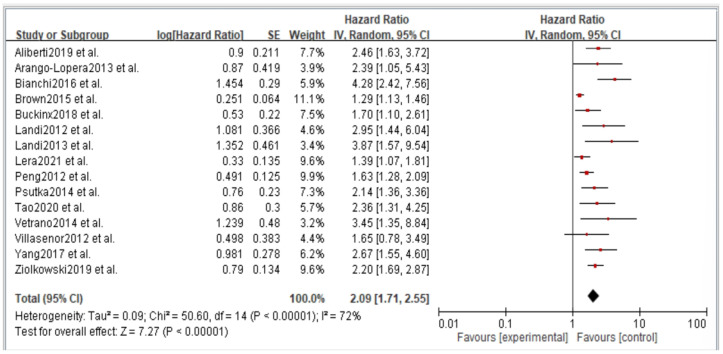
Summary estimates for the sarcopenia status compared to mortality outcome.

Figure 2, Figure 3, Figure 4, Figure 5 and Figure 6 showed that, when compared to robust individuals, the risk of injury events for older individuals with sarcopenia was significantly higher in fall (HR = 1.93, CI: 1.29–2.87), fractures (HR = 2.25, CI: 1.77–2.86), hospital admissions (HR = 1.52, CI: 1.28–1.80), disability (HR = 2.74, CI: 1.73–4.34), and death (HR = 2.09, CI: 1.71–2.55). In consideration of the negative impact of sarcopenia on the subsequent health of older adults, professional nursing personnel should assess older adults for sarcopenia as early as possible and propose relevant care policies to further reduce negative health impacts. (Figure 2, Figure 3, Figure 4, Figure 5 and Figure 6).

**Table 2:** Newcastle–Ottawa scale quality assessment for prospective cohort studies.

First Author	Selection				Comparability	Outcome			
	Representativeness of the Exposed Cohort	Selection of the Non-Exposed Cohort	Ascertainment of Exposure	Demonstration that Outcome of Interest was not Present at the Start of the Study	Comparability of Cohorts on the Basis of the Design or Analysis	Assessment of Outcome	Was Follow-Up Long Enough for Outcomes to Occur?	Adequacy of Follow-up of Cohorts	Overall Quality Score (Maximum = 9)
da Silva Alexandre et al. [10]	★	★	★	★	★★	★	★	★	9
Aliberti et al. [11]	★	-	★	★	★★	★	★	★	8
Arango-Lopera et al. [41]	★	★	★	★	★★	★	★	★	9
Benjumea et al. [30]	★	★	★	★	★★	★	★	★	9
Bianchi et al. [40]	★	★	★	★	★★	★	★	★	9
Brown et al. [42]	★	-	★	★	★★	★	★	★	8
Buckinx et al. [31]	★	★	★	★	★★	★	★	★	9
Cawthon et al. [38]	★	★	★	★	★★	★	★	★	9
Gariballa et al. (2013)	★	★	★	★	★★	★	★	★	9
Cawthon et al. [46]	★	-	★	★	★★	★	★	★	8
Chalhoub et al. [37]	★	★	★	★	★★	★	★	★	9
Harris et al. [21]	★	★	★	★	★★	★	★	★	9
Henwood et al. [22]	★	★	★	★	★★	★	★	★	9
Landi et al. [23]	★		★	★	★★	★	★	★	8
Lera et al. [43]	★	★	★	★	★★	★	★	★	9
Lim et al. [32]	★		★	★	★★	★	★	★	8
Landi et al. [14]	★	★	★	★	★★	★	★	★	9
Matsumoto et al. [33]	★	★	★	★	★★	★	★	★	9
Peng et al. [44]	★	★	★	★	★★	★	★	★	9
Pérez-Zepeda et al. [25]	★		★	★	★★	★	★	★	8
Psutka et al. [45]	★	★	★	★	★★	★	★	★	9
Scott et al. [36]	★	★	★	★	★★	★	★	★	9
Sjoblom et al. [16]	★	★	★	★	★★	★	★	★	9
Tanimoto et al. [18]	★		★	★	★★	★	★	★	8
Tao et al. [19]	★	★	★	★	★★	★	★	★	9
Vetrano et al. [20]	★	★	★	★	★★	★	★	★	9
Villasenor et al. [5]	★	★	★	★	★★	★	★	★	9
Woo et al. [6]	★		★	★	★★	★	★	★	8
Yang et al. [7]	★	★	★	★	★★	★	★	★	9
Yu et al. [3]	★	★	★	★	★★	★	★	★	9
Ziolkowski et al. [2]	★	★	★	★	★★	★	★	★	9

★ present one score.

## 4. Discussion

The WHO [9] has indicated that the prevention of sarcopenia is one of the important indicators for the successful aging of older adults. This study was the first study to perform an overall analysis of the future health effects (including falls, fractures, hospitalization, disability, and death) of sarcopenia on older adults over the age of 65. The results of this evidence-based study showed that, compared with older adults without sarcopenia, older adults with sarcopenia have a higher risk of experiencing negative health outcomes, such as falls, fractures, hospitalizations, disability, and death. Overall, the meta-analysis demonstrated that these studies indicate that sarcopenia is the major factor of the increased risk for all injury events. Therefore, medical and nursing personnel must pay attention to the older adults experiencing sarcopenia, as once the symptoms occur, it may start to affect their future health, cause a significant impact on their future health, and even result in death. Chang et al. [8] indicated that due to sarcopenia, older adults may easily experience subsequent injury events, which may create a burden for individuals, families, caregivers, and society. It has been estimated that the expenses arising from falls, fractures, and hospitalizations caused by sarcopenia in older adults per year in the United States are approximately USD 11.8 billion to USD 26.2 billion [47]. Therefore, medical and nursing personnel must assess the sarcopenia state of older adults as early as possible to provide care policies and reduce and alleviate the further occurrence of injury events.

This study collected 38 studies investigating a total of 167,930 older subjects to study the effects of sarcopenia on the prospective health of older adults, including falls, fractures, hospitalizations, disability, and death. This study found that the mean follow-up time for subsequent injury events was 8.75 years (SD = 2.08). However, there were significant differences in the follow-up time scope among various studies. The follow-up time for death was the longest, with a mean of 6.17 years (SD = 2.83), while the follow-up time for falls was the shortest, with a mean of 1.7 3 years (SD = 0.15). Chu et al. [48] indicated that for the injury events caused by sarcopenia, the poor health status varies with the health status of older adults and may experience a slow process. Therefore, long-term follow-up is required during the assessment.

There were several features of note in this study. This study was the first to implement a systematic review and meta-analysis to analyze the prediction of sarcopenia among older adults with subsequent negative health outcomes. Therefore, the research results have an important reference value. Nevertheless, there were still some limitations in this study. Firstly, the meta-analysis showed that the assessment criteria for sarcopenia were different among various studies, which might have resulted in deviations in the statistical analysis. Secondly, there were significant differences in the follow-up times of various studies. The shortest follow-up time was one year, while the longest one was 12 years, which might have affected the prediction of the risk of negative outcomes. Lastly, although most of the studies presented controlled intervening variables, this study still could not fully overcome the individual intervening factors. As a result, the estimation of consistency may have been affected. However, although the aforementioned limitations affected the conclusions and interferences of the meta-analysis in this study, the study findings are worthy of reference by professional medical and nursing personnel as the basis for further development of care strategies in the future.

### 4.1. Conclusions

Sarcopenia is an important issue in older adults’ care. Evidence-based studies have shown that sarcopenia is highly correlated with subsequent injury events, including falls, fractures, hospitalization, disability, dementia, and death. The differences in sarcopenia criteria usually will not result in different interpretation results. Therefore, medical and nursing personnel must assess the sarcopenia state of older adults in a timely manner and provide effective improvement schemes to reduce the further risk of sarcopenia in older adults.

### 4.2. Clinical Implications

Evidence-based studies have verified that there is a high prediction of subsequent injury events for older adults with sarcopenia. Medical and nursing personnel should make the best use of sarcopenia assessment criteria early on to help older adults receive sarcopenia screening and detect high-risk subjects. In particular, compared with older adults without sarcopenia, older adults with sarcopenia are more likely to experience subsequent injury events, such as fractures, hospitalizations, and death. Therefore, medical and nursing personnel are recommended to pay more attention to the health status of older adults with sarcopenia, as well as designing holistic care schemes to effectively reduce the risk of subsequent injury events and improve the quality of life of older adults.

## Figures and Tables

**Table 1:** Characteristics of the included studies for meta-analysis.

No.	First Author	Population	Sarcopenia Criteria	Sample Size	Sex	Age	Length of Follow Up	HR (95% CI)	Variable Adjusted
Fall									
1	Benjumea et al. [30]	Clinic	EWGSOP	534	F	75	12 years	1.06 (0.98–1.14)	None
2	Buckinx et al. [31]	Nursing home	EWGSOP	662	F/M	≥85	1-year	1.70 (1.10–2.92)	None
3	Henwood et al. [22]	Nursing home	EWGSOP	58	F/M	75–95	18 months	0.74 (0.34–1.63)	None
4	Lim et al. [32]	Hospitalized patients	AWGS	147	F	65	2.5 years	2.354 (1.177–4.709)	None
5	Matsumoto et al. [33]	Hospitalized patients	EWGSOP	162	F/M	60	2 years	7.68 (1.41–41.77)	Adjusted for age, sex, body mass index, previous falls, locomotive syndrome and visual analog scale.
6	Mori and Tokuda [34]	Community-dwelling	AWGS	331	F	≥70	2-year	3.03 (1.01–9.09)	None
7	Schaap et al. [35]	Community-dwelling	EWGSOP	496	F/M	75	3 years	1.29 (0.89–1.87)	Adjusting for age, sex, and total body fat
8	Scott et al. [36]	Community-dwelling	EWGSOP	101/1575	M	≥70	2 years	2.15 (1.58–2.94)	Adjusted for age, income, living alone, number of comorbidities, smoking status, psychotropic and corticosteroid use, history of fracture, physical activity and 25(OH)D.
9	Sjoblom et al. [16]	Community-dwelling	EWGSOP	590	F	65–72	1-year	3.3 (1.6–7.0)	Adjusted for: age, body mass index (BMI), physical activity and hormone therapy (HT).
10	Woo et al. [6]	Community-dwelling	AWGS	2848	F/M	65	1-year	1.59 (1.02–2.49)	None
Fracture									
1	Chalhoub et al. [37]	Community-dwelling	EWGSOP	5544	F/M	65	2 years	1.19 (0.65–2.17)	Adjusted Age
2	Cawthon et al. [38]	Community-dwelling	EWGSOP	1516	F/M	70–80	3 years	1.68 (0.74–3.81)	None
3	Chen et al. [39]	Hospitalized patients	EWGSOP	990	F/M	60	1-year	2.03 (1.29–3.19)	None
4	Harris et al. [21]	clinical centers	EWGSOP	10,937	F	63	3 years	0.85 (0.64–1.12)	Adjusted for age, clinic, and race.
5	Schaap et al. [35]	Community-dwelling	EWGSOP	496	F/M	75	10 years	0.94 (0.54–1.64)	adjusting for age, sex, and total body fat
6	Scott et al. [36]	Community-dwelling	EWGSOP	106/1575	M	≥70	2 years	1.06 (0.51–2.18)	Adjusted for age, income, living alone, number of comorbidities, smoking status, psychotropic and corticosteroid use, history of fracture,
7	Sjoblom et al. [16]	Community-dwelling	EWGSOP	590	F	65–72	1-year	2.60 (1.84–3.68)	Adjusted for: age, body mass index (BMI), physical activity and hormone therapy(HT)
8	Yu et al. [3]	Community-dwelling	AWGS	4000	F/M	65	1.5 years	4.74 (2.71–8.28)	None
Hospitalization									
1	Aliberti et al. [11]	Hospitalized patients	EWGSOP	203/665	F	80	1-year	1.53 (1.16–2.04)	adjusted for age, sex, race, income
2	Bianchi et al. [40]	Community-dwelling	EWGSOP	55/538	F	65–94	2 years	1.57 (1.03–2.41)	
3	Cawthon et al. [38]	Community-dwelling	EWGSOP	421/1516	F/M	70–80	3 years	1.27 (0.85–1.90)	adjusted -Age
4	Gariballa (2013)	Hospitalized patients	EWGSOP	432	F	≥65	180 days	0.53 (0.32–0.87)	
5	Henwood [22]	Nursing home	EWGSOP	58	F/M	75–95	18 months	1.25 (0.73–2.14)	
6	Pérez-Zepeda et al. [25]	Hospitalized patients	EWGSOP	172	F/M	≥70	1-year	0.92 (0.62–1.37)	
7	Yang et al. [7]	Hospitalized patients	AWGS	313	M	60	3 years	1.82 (1.28–2.59)	
Functional disability									
1	da Silva Alexandre et al. [10]	Community-dwelling	EWGSOP	328/478	F/M	60	4-year	5.26 (0.84 –2.84)	None
2	Benjumea et al. [30]	Clinic	EWGSOP	144/534	F	75	12 years	2.03 (1.18–3.50)	None
3	Bianchi et al. [40]	Community-dwelling	EWGSOP	36/538	F	65–94	2 years	4.78 (1.84–12.7)	adjusting for Age and Sex
4	Tanimoto et al. [18])	Community-dwelling	EWGSOP	743	F/M	65	2-year	10.4 (1.8–59.8)	adjusted for age and body mass index
5	Woo et al. [6]	Community-dwelling	AWGS	4000	F/M	65	4-year	2.04 (1.32–3.17)	adjusted for age, education, COPD, diabetes mellitus, hypertension, heart disease, current smoker, MMSE, and depression
Mortality									
1	Aliberti et al. [11]	In-hospital patients	EWGSOP	203/665	F	80	1-year	2.46 (1.63–3.72)	adjusted for age, sex, race, income
2	Androga et al. [12]	In-hospital patients	EWGSOP	1082	M	65	5 years	1.32 (1.06–1.66)	None
3	Arango-Lopera et al. [41]	Community-dwelling	EWGSOP	345	F/M	78	5 years	2.39 (1.05–5.43)	None
4	Bianchi et al. [40]	Community-dwelling	EWGSOP	55/538	F	65–94	2 years	4.28 (2.42–7.59)	None
5	Brown et al. [42]	Community dwelling	EWGSOP	4425	F/M	≥60	6 years	1.29 (1.13–1.47)	None
6	Buckinx et al. [31]	Nursing home	EWGSOP	662	F/M	≥85	1-year	1.70 (1.10–2.92)	None
7	Gariballa (2013)	In-hospital patients	EWGSOP	258	F/M	≥65	180 days	0.45 (0.21–0.97)	None
8	Henwood [22]	Nursinghome	EWGSOP	58	F/M	75–95	18 months	0.81 (0.33–1.98)	None
9	Landi et al. [14]	Community dwelling	EWGSOP	197	F/M	80–85	300 days	2.95 (1.44–6.04)	None
10	Landi et al. [23]	Nursing home	EWGSOP	146	F/M	>70	300 days	3.87 (1.57–9.54)	None
11	Lera et al. [43]	community-dwelling	EWGSOP	2311	F/M	≥60	5-year	1.39 (1.07–1.82)	adjusting for age, sex, nutritional status, and number of chronic diseases,
12	Pereira et al. [24]	In-hospital patients	EWGSOP	287	M	≥70	40 months.	3.02 (1.30–7.05)	None
13	Peng et al. [44]	In-hospital patients	EWGSOP	1953	F/M	65	2 years	1.63 (1.28–2.07)	None
14	Psutka et al. [45]	In-hospital patients	EWGSOP	205	F/M	72	2 years	2.14 (1.24–3.71)	None
15	Tandon et al. [17]	Community-dwelling	EWGSOP	258	F/M	≥18	2 years	2.36 (1.23–4.53)	None
16	Tao et al. [19]	In-hospital patients	EWGSOP	427	M	80	32 months	2.36 (1.31–4.24)	None
17	Vetrano et al. [20]	In-hospital patients	EWGSOP	770	F	82	1 year		adjusting for Age- and Gender
18	Villasenor et al. [5]	In-hospital patients	EWGSOP	75/471	F	≥50	270 days	1.65 (0.78–3.52)	adjusted-Age
19	Yang et al. [7]	In-hospital patients	AWGS	313	M	60	3 years	2.67 (1.55–4.60)	None
20	Ziolkowski et al. [2]	Community-dwelling	EWGSOP	534	F	≥60	2 years	2.20 (1.69–2.86)	adjustment for age, sex, race/ethnicity, physical activity, smoking status, diabetes, cancer, liver disease, ardiovascular disease, education, and income

## Data Availability

Data available on request due to privacy/ethical restrictions.
