# Correction: Su et al. The Relationship between Sarcopenia and Injury Events: A Systematic Review and Meta-Analysis of 98,754 Older Adults. *J. Clin. Med.* 2022, *11*, 6474

**DOI:** 10.3390/jcm14093084

**Published:** 2025-04-29

**Authors:** Yu-Chen Su, Shu-Fang Chang, Hsiao-Chi Tsai

**Affiliations:** 1 Department of Nursing, College of Nursing, National Taipei University of Nursing and Health Sciences, 365 Ming Te Road, Pei-Tou, Taipei 112303, Taiwan; 2 Cardinal Tien Hospital, No.15, Chezi Rd., Xindian Dist., New Taipei City 112303, Taiwan

## Text Correction

1. There was an error in the original publication [1]. There was an author’s typographical error in statistical values. A correction has been made to the Abstract.

**Abstract:** The main purpose of this study was to investigate the relationship between sarcopenia and injury events (falls, fractures, hospitalization, disability, and death). This study systemically searched the literature from Embase, PubMed, MEDLINE, CINAHL, and Cochrane Library and analyzed the collected literature using the random effects model to demonstrate the relationship between sarcopenia and injury events. This study followed the guidelines of the Preferred Reporting Items for Systematic Reviews and Meta-Analyses (PRISMA) and collected a total of 35 prospective studies. The results showed that, when compared to robust individuals, the risk of injury events for older individuals with sarcopenia was significantly higher for fall (HR = 1.93, CI: 1.29–2.87), fractures (HR = 2.25, CI: 1.77–2.86), hospital admissions (HR = 1.52, CI: 1.28–1.80), disability (HR = 2.74, CI: 1.73–4.34), and death (HR = 2.09, CI: 1.71–2.55). In consideration of the negative impact of sarcopenia on the subsequent health of older adults, professional nursing personnel should assess older adults for sarcopenia as early as possible and propose relevant care policies to further reduce negative health impacts.

2. In the original publication [1], there was an Author’s typographical error in statistical values and figures. A correction has been made to “3.3. Association between Sarcopenia and Injury Events”.

### 3.3. Association between Sarcopenia and Injury Events

Figures 2–6 showed that, when compared to robust individuals, the risk of injury events for older individuals with sarcopenia was significantly higher in fall (HR = 1.93, CI: 1.29–2.87), fractures (HR = 2.25, CI: 1.77–2.86), hospital admissions (HR = 1.52, CI: 1.28–1.80), disability (HR = 2.74, CI: 1.73–4.34), and death (HR = 2.09, CI: 1.71–2.55). In consideration of the negative impact of sarcopenia on the subsequent health of older adults, professional nursing personnel should assess older adults for sarcopenia as early as possible and propose relevant care policies to further reduce negative health impacts. (Figures 2–6).

## Error in Figure

In the original publication [1], there was a mistake in Figures 2–6 as published. The corrected Figure 2, Figure 3, Figure 4, Figure 5 and Figure 6 appear below.

The authors state that the scientific conclusions are unaffected. This correction was approved by the Academic Editor. The original publication has also been updated.

## Figures and Tables

**Figure 2:**
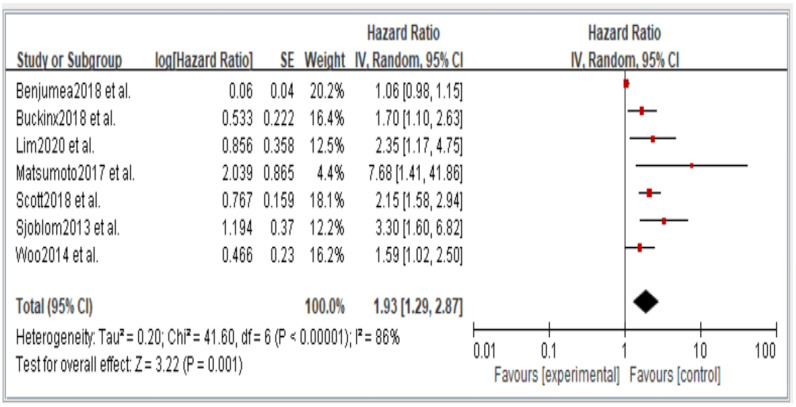
Summary estimates for the sarcopenia status compared to fall outcome.

**Figure 3:**
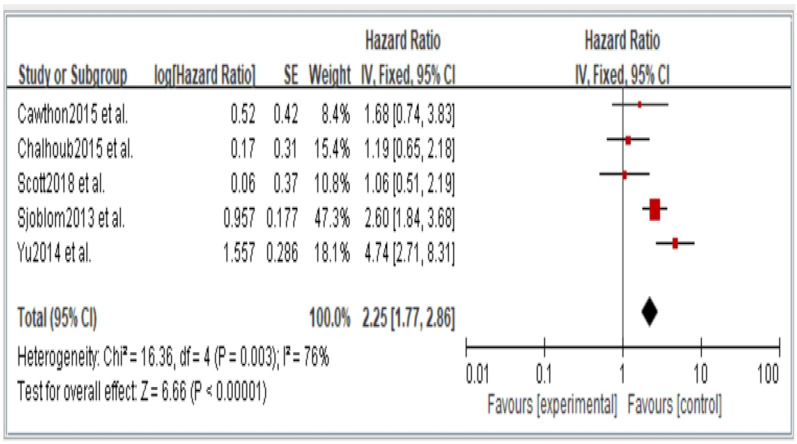
Summary estimates for the sarcopenia status compared to fracture outcome.

**Figure 4:**
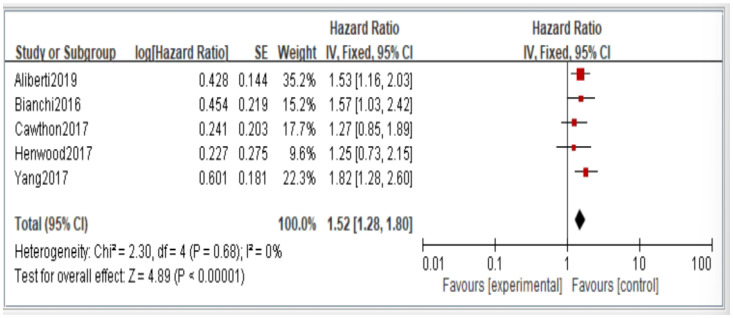
Summary estimates for the sarcopenia status compared to hospitalization outcome.

**Figure 5:**
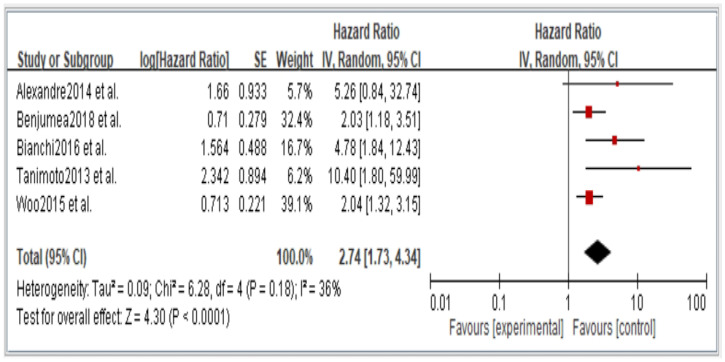
Summary estimates for the sarcopenia status compared to disability outcome.

**Figure 6:**
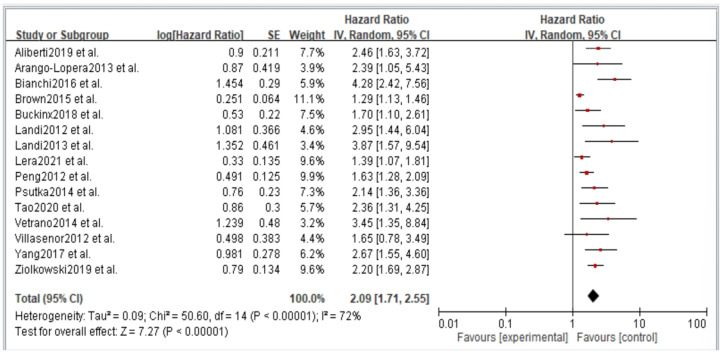
Summary estimates for the sarcopenia status compared to mortality outcome.
